# Supplementary material for: Are carbon emissions trading and green financial instruments synergistic? -Comprehensive quantitative research based on content analysis
Source: PLoS One. 2024 Mar 7;19(3):e0298601. doi: 10.1371/journal.pone.0298601 (PMC10919601; doi:10.1371/journal.pone.0298601)
Supplement: S1 Data — (ZIP) [file pone.0298601.s001.zip › Data/地级市Z_F工作报告.zip!/╜¡╬≈/╛░╡┬╒≥╩╨/└┤╫╘╣1⁄2╓┌║┼í╛┬φ┐╦╩2╛▌═°í┐_2017.docx]

各位代表：

现在，我代表市人民政府，向大会报告政府工作，请予审议，并请各位政协委员和其他列席人员提出意见。

过去五年，在省委、省政府和市委的坚强领导下，我们坚持稳中求进工作总基调，主动适应经济发展新常态，突出城市、产业和文化，全面实施工业、城建、招商“三大战役”，全市经济社会发展取得了可喜的进步。

经济总量不断做大。2015年，全市生产总值772亿元，比2010年增长62.9%；财政总收入112亿元，增长109.9%；固定资产投资691亿元，增长101.4%；社会消费品零售总额268亿元，增长91.1%；农业总产值88亿元，增长22.1%；服务业增加值277亿元，增长56.9%；规模以上工业增加值271亿元，增长85.7%。粮食生产“十二连丰”，乐平现代农业示范区晋升为国家级，“浮梁茶”入选全省五大茶叶品牌。主营业务收入超亿元企业159户，昌飞、焦化突破百亿元；华意压缩新600项目一期竣工投产，成为全球最大的家用压缩机制造商；天新药业、宏柏化工等企业主要产品市场占有率国际领先；世龙实业、富祥药业首发上市。我市成为全国首批服务业综合改革试点城市；大学生陶瓷创业孵化园被评为国家级示范基地；成功举办每年一届的瓷博会；连续三年跻身全国电商百佳城市。

产业格局不断优化。高新区成为“双国家级”直升机产业基地；陶瓷工业园获批国家新型工业化陶瓷制品产业示范基地；乐平工业园获批国家级精细化工高新技术产业化基地。陶瓷产业总产值337亿元，增长1.1倍；陶溪川核心启动区、名坊园一期、御窑景巷等陶瓷文化产业项目建设完成；以乐华、金意陶、欧神诺为代表的建卫陶瓷不断做大；新增“红叶”、“昌南”等4个“中国驰名商标”，陶瓷股份公司获评中国陶瓷行业最具竞争力十强企业。航空产业总产值154亿元，增长3.7倍；拥有昌飞、北通航、德利3家整机生产企业和39家零部件配套企业，形成了“产能百架、产值百亿”的航空产业集群。汽车产业总产值100亿元，增长35.1%；北汽集团顺利重组昌汽，整车项目一期竣工投产，Q25、Q35成功上市。旅游产业总收入261亿元，增长2.9倍；入选“中国最具魅力文化旅游城市”和“中国景区国际影响力20强”；古窑成功创建5A景区，新增御窑、皇窑、怪石林3个4A景区；浮梁县被评为全国休闲农业与乡村旅游示范县。

城乡面貌不断改善。完成城市总体规划修编和历史文化名城保护规划编制，中心城区控规覆盖率80%。规划建设赣东北综合物流园、昌南拓展区、高铁商务区三大功能新区，绿地陶瓷文化旅游城全面开工，建成区面积扩大到86平方公里。九景衢铁路建设顺利推进，昌景黄高铁列入国家“十三五”路网规划。浯溪口水利枢纽主体工程年底竣工。建成全省首条城市地下综合管廊。西河水系综合治理、珠山路改造、三宝国际瓷谷、陶艺街等重大项目加快推进。贯彻落实党中央、国务院领导重要批示精神，有序推进以御窑厂遗址为龙头的大遗址保护和世界文化遗产申报工作，御窑遗址博物馆开工建设。统筹推进以陶溪川为核心的老工厂文化创意产业项目。全面启动“五城同创”，突出“整齐、整洁、整治”，严厉打击违法用地和违章建筑，围绕交通拥堵、户外广告、“门前三包”等16个方面整治市容市貌，蝉联“全国文明城市提名城市”。新农村建设深入开展，在全省率先推进城乡供水一体化，乐平市、浮梁县、昌江区获评全省新农村建设、清洁工程等先进单位。全市森林覆盖率65.7%，建成区绿化覆盖率52.1%；生态环境状况连续五年全省第一，浮梁县成功创建国家级生态县。

改革开放不断深化。主动融入“一带一路”、长江经济带等国家战略，成为联合国海陆丝绸之路城市联盟首批创始成员，与国外20多个产瓷城市建立友好关系；积极参加米兰世博会、赣台会、赣港会等经贸合作活动。利用外资增长51.8%，引进内资增长228%，出口增长199%；华意压缩、黑猫炭黑等9家企业实现“走出去”目标。完成部分行政区划调整和新一轮政府机构改革，精简规范行政权力事项4874项，精简率60%以上，市场监管体制改革走在全省前列；统筹推进了城管体制、商事制度、国资国企、农业农村、国有林场、警务机制、公务用车等改革。

社会事业不断进步。城乡居民可支配收入快速增长，城乡低保标准大幅提升；城镇新增就业累计18.2万人，发放创业担保贷款21亿元；五项保险参保总人数176.7万人次；建成保障房1.6万套，棚户区改造开工4万户，2016年棚改面积超过“十二五”时期任务总和。公交、出租车全部更新。4G网络覆盖城乡。信访形势总体稳定，建成21个专项矛盾纠纷调处中心；社会治安好转率连续5年全省第一；连续14年未发生重特大安全生产事故。各级各类教育事业协调发展，景德镇陶瓷学院更名为景德镇陶瓷大学，景德镇高专升格为景德镇学院，老年大学新教学楼投入使用。直升机研制荣获全国科技进步一等奖。院士工作站建设实现“零突破”，全国唯一的陶瓷知识产权快速维权中心获批设立。在全省率先实现县级公立医院综合改革全覆盖。中国陶瓷博物馆建成开馆。市政府与故宫博物院签订合作框架协议。获评“世界手工艺与民间艺术之都”并加入全球创意城市网络。成功承办全国第9个文化遗产日主场城市活动。被评为“全国群众体育先进单位”，实现全国双拥模范城“六连冠”。取得了抗击2012年“8·10”和今年“6·19”特大暴雨洪灾的重大胜利，有效保障了G20峰会、全国综治工作“南昌会议”等重要会议的顺利召开。同时，其他各项社会事业都取得了新进步。

政府自身建设不断加强。深入开展了“转作风、提效能、促发展”活动、党的群众路线教育实践活动、“三严三实”专题教育和“两学一做”学习教育；严格落实中央八项规定精神，驰而不息改进作风，“三公”经费大幅压减；坚持依法行政，自觉接受人大依法监督和政协民主监督，认真办理人大代表建议490件、政协委员提案780件；加大行政监察审计监督力度，全面推行政务信息公开，政府公信力和执行力进一步提高。

各位代表，这些成绩是省委、省政府和市委领导下全市人民辛勤劳动的结果。在此，我代表市人民政府，向全市广大干部群众，向各位人大代表、政协委员、各民主党派、工商联、无党派和社会各界人士，向驻景各单位、解放军、武警官兵、公安干警和离退休老同志，向所有关心、支持、参与景德镇建设发展的朋友们，表示衷心的感谢并致以崇高的敬意!

当然，我们也清醒地认识到，我市经济总量偏小、发展质量仍然不高，面临做大总量和提升质量的双重压力；特色产业的集聚放大效应还未充分显现；城市建设和管理、创意文化发展还面临许多新课题；社会事业发展水平与群众新期盼仍有差距；少数公职人员不作为、不会为，存在庸政懒政怠政等现象。对此，我们将以科学务实的态度、坚决有力的措施，切实加以改进和解决。

各位代表，今后五年政府工作的指导思想是：高举中国特色社会主义伟大旗帜，以邓小平理论、“三个代表”重要思想和科学发展观为指导，深入贯彻习近平总书记治国理政新理念新思想新战略，全面落实习近平总书记对江西工作“新的希望、三个着力、四个坚持”的总体要求，按照“五位一体”总体布局和“四个全面”战略布局，以“五大发展理念”为引领，以“提质翻番、率先小康”为目标，全力实施“三个五”战略行动，为“复兴千年古镇、重塑世界瓷都、保护生态家园、建设旅游名城，打造一座与世界对话的城市”而努力奋斗。

今后五年政府工作的总体要求是：坚持发展为先、实干为本、创新为要，正确处理好继承与创新的关系。继承，就是要保持工作的连续性，一届接着一届干，持之以恒抓好产业升级、城乡建设、民生改善等各项工作，不断提升景德镇的综合竞争力；创新，就是要主动适应新形势、新要求，破解新难题、培育新优势，锐意改革创新，全面扩大开放，全力保障“三个五”战略的顺利实施。

今后五年政府工作的主要目标是：“两个翻番”、“位次前移”、“率先小康”，即：与2010年相比，人均生产总值和城乡居民可支配收入翻番；地区生产总值年均增长8.5%以上，财政总收入年均增长10%以上，固定资产投资年均增长12%以上，主要经济指标增速在全省位次前移；现行标准下贫困人口实现脱贫，在全省率先建成全面小康社会。

为此，我们将重点做好以下工作。

一、坚持发展为先，不断提高经济综合实力

未来五年，我们将牢牢把握发展第一要务，以项目为抓手、以园区为平台，加快产业转型升级，做大做强支柱产业群，不断增强发展的实力。

壮大“3+1”产业支柱。苦干五年，为未来打造陶瓷、航空、汽车、旅游四大千亿产业奠定坚实基础。（1）加快陶瓷产业升级。陶瓷是景德镇的立市之本。弘扬“工匠精神”，坚持传承传统陶瓷和创新现代陶瓷融合发展，大力发展手工制瓷、高技术陶瓷；科学制定陶瓷产业集群规划，搭建陶瓷公共技术平台，深化行业体制改革，培育陶瓷龙头企业，实施陶瓷品牌战略，依法管理陶瓷市场，运用“互联网+陶瓷”理念，促进陶瓷产业向高端化、品牌化、国际化、规模化发展，打造陶瓷产业升级版。力争到2020年，新增规模以上陶瓷企业30家，实现产业增加值120亿元、上市企业零突破。（2）抢占航空产业高地。航空是景德镇的未来希望。抢抓国家加快军民融合、通用航空和特色小镇发展的重要战略机遇，坚持“创新引领、军民融合、产融融合、产城融合”，高起点编制航空产业规划，努力建设国内一流、国际知名的航空特色小镇。力争实现航空产业增加值180亿元。（3）推动汽车产业集聚。汽车是景德镇的现实潜力。不断深化与北汽的合作，完善汽车产业链，发展新能源汽车，力争早日建成集整车与发动机产、供、销、研于一体的汽车产业制造基地，实现“产销整车65万辆、发动机30万台”、产业增加值100亿元的目标。（4）提升旅游产业水平。旅游是景德镇的财富之源。创建国家全域旅游示范区，促进旅游与文化、陶瓷、生态、互联网等深度融合。完善瑶里、古县衙、洪岩仙境等景区建设，打造一批体现景德镇独特魅力的重点旅游景区。加大旅游宣传推介力度，推进国际旅游集散中心、休闲度假中心等配套设施建设，加强与周边地区广泛合作，努力成为“国际知名、国内一流”旅游目的地。力争新增2家5A级景区，实现旅游产业增加值150亿元。

做大文化创意产业。发挥陶瓷文化底蕴深厚的优势，延续近现代陶瓷工业生产文明，继续推进陶溪川、名坊园、三宝国际瓷谷等陶瓷文化创意产业集聚区建设，打造一批文化产业园区、特色文化街区、文化示范基地。延伸文化产业链，壮大以陶文旅集团为龙头的一批文化产业集团，推动中小型文化企业集群发展，形成一批具有自主知识产权的产品。力争文化创意产业实现增加值100亿元。同时，加快物流业发展，促进与产业体系、电子商务、现代交通的对接与配套，推进电商产业园、电商示范基地建设。大力发展云计算、智能制造等新兴产业和业态。促进商业银行以及各类金融机构、金融业态的发展和聚集。积极发展健康养老、体育休闲、家政服务等生活性服务业。

推动现代农业发展。以工业化理念谋划农业，不断加大对农业农村的投入，推动农业特色化、产业化、生态化和现代化发展。加强基本农田保护和水利基础设施建设，推进土地整治、中低产田改造和高标准绿色生态农田建设，开展粮食高产创建和绿色增产模式攻关，稳步提高粮食综合生产能力和效益。加快推进以乐平全国现代农业示范区、浮梁全国绿色有机食品示范区为代表的“一县一园”建设，促进蔬菜、茶叶、油茶、花卉苗木等特色产业集群化发展。实施“一村一品”工程，重点推进产值超亿元的特色产业村建设，形成“村有特色、乡有规模、业有龙头”的区域特色经济发展格局。力争农业增加值60亿元，乐平蔬菜和浮梁茶叶产业跻身全国先进行列。

推进重大项目建设。项目是经济发展的主抓手。加快实施一批重大产业项目，全面建设“航空小镇”，完成昌飞总装园、北通航江直公司产业基地、德利直升机项目建设；谋划建设“汽车小镇”，完成昌河汽车研发中心和汽车配套产业园建设，尽早启动北汽昌河新基地二期项目；加快推进红叶新厂、昌江百里风光带、陶源谷等项目建设。加快实施一批重大基础设施项目，完成浯溪口水利枢纽工程建设；建成九景衢铁路，打造景德镇北站综合交通枢纽，力争明年底开工建设昌景黄高铁，启动六安景铁路前期工作，争取皖赣线改线工程早日开工；规划建设新机场；适时启动景德镇港综合码头建设；推进地下综合管廊国家试点，打造民生工程、百年工程、创新工程。加快实施一批重大社会事业项目，以御窑厂遗址为核心，打造具有世界影响力的“景德镇御窑厂国家考古遗址公园”，把御窑遗址博物馆建成具有国际影响的文化地标，力争成功申报世界文化遗产；完善公共图书馆、博物馆、档案馆、方志馆等设施；新建景东游泳馆、综合训练馆，完善市体育场，改造射击馆。同时，加快实施城市公园、农贸市场、垃圾中转站、停车场、公厕等一批事关老百姓切实利益的民生工程项目。

激活县域发展活力。县域是经济发展的主力军。要为县域经济“松绑放权”，促进各县（市、区）竞相发展、错位发展、特色发展。支持乐平市“建设特色鲜明的赣东北经济、开放、活力、生态、和谐、廉洁明珠”；浮梁县“打造生态样板，建设旅游胜地，做美山水城市，建成对话世界的后花园”；昌江区“建设重点产业集聚区、创新创业示范区、生态文明样板区、文化旅游拓展区和幸福都市生活区，做靓瓷都门户”；珠山区“建设经济繁荣、城市靓丽、古韵悠长、和谐宜居的历史文化名城标志性城区”。

打造园区经济高地。园区是经济发展的主战场。坚持产城融合、集约发展、合作共赢，创新园区建设和管理机制，完善功能配套，推动产业层次攀升；加速做大经济总量，努力将园区建设成为技术、人才、资金、服务密集的高端产业聚集区，实现在全省园区中进位赶超。支持高新区依托昌飞、华意等龙头企业，做大做强支柱产业，力争早日跻身千亿园区，建成我市经济发展的新增长极、国内有竞争力的高新技术产业园区和现代化园林化新城区；支持陶瓷工业园区做优做强陶瓷产业，依托北汽昌河新基地，发展汽车及配套产业，力争早日跻身千亿园区，成为全市陶瓷产业、文化、旅游三个地标；支持乐平工业园区建成全国具有影响力的精细化工集散中心。

二、坚持统筹城乡，促进区域协调发展

未来五年，我们将坚持实施城乡一体化战略，扎实推进新型城镇化，协调区域发展，不断增强城市的张力。

做优中心城市。（1）科学规划城市。推进全域“多规合一”，推动各类规划相互衔接和有效融合；科学规划设计城市重要节点和核心区域，严控规划“五线”，合理划定边界，优化空间布局，实现中心城区控制性详规全覆盖；加快规划编制成果法制化进程，切实维护规划权威。（2）高效建设城市。加快赣东北综合物流园、高铁商务区和昌南拓展区核心区等功能新区建设。继续实施“老街区、老厂区、老窑址”保护，加快恢复老城区历史风貌。结合名城保护、产业发展、生态修复，全力推进城市棚户区改造。（3）精心管理城市。深化城管体制改革，强化街道、社区公共服务职能，落实属地管理责任，提升城市管理数字化、网格化、精细化水平；继续推进“五城同创”，实现创建工作常态化、制度化、长效化，力争蝉联省级文明城市，晋级全国文明城市、国家卫生城市、国家森林城市；继续严厉打击“两违”，合理利用存量土地，最大化发挥土地效益；通过探索设立城建发展基金、推进PPP等模式，广泛吸引社会资本参与城市建设。

建设美丽乡村。瓷都要美，农村必须美。充分发挥生态优势和产业特色，保留乡村风貌、乡土味道，按照“整整齐齐、干干净净、漂漂亮亮、和谐宜居”的要求，大力推进新农村建设。继续推进扩权强镇改革和试点工作，加强重点镇基础设施和公共服务能力建设，提升小城镇在联系城乡中的关键作用。因地制宜，建设一批独具韵味、充满魅力的特色小镇。按照“限高、限大、治乱、打违”的要求，加强小城镇、村庄和农民建房的规划管理。大力整治农村人居环境，基本实现垃圾处理城乡一体化覆盖，有序开展农村生活污水处理。推进乡风文明建设，营造科学健康、文明向上、生机勃勃的农村社会风貌。

优化城乡路网。完善城市主干路网，疏通道路微细循环，合理贯通城市断头路，加快南面绕城快速通道建设，启动新跨江大桥和高架桥的论证规划工作；积极对接高速路网，力促景鹰和德昌高速连接线早日实施，开通月亮湖景南高速出口，增设德昌高速乐平工业园出入口。完善全市公路骨架网，实施351国道、206国道升级改建，形成快速过境大通道。力争到2020年，90%的国道、60%的省道达到二级公路以上标准，乡镇通三级公路，行政村通班车，25户以上自然村通水泥路。

三、坚持绿色发展，建设美丽宜居生态家园

未来五年，我们将坚定实施可持续发展战略，坚持生态立市、绿色发展，不断增强发展的持久力。

加强生态治理。落实大气污染行动计划，抓好主要污染物减排，积极发展清洁能源，推动重点行业能效提升和节能减排升级改造，保持空气优良天数位居全省前列。推进城市污水处理及配套管网建设。落实“河长制”，开展以昌江河、乐安河水域为重点的清河行动，实现“河畅、水清、岸绿、景美”。继续实施乐安河流域重金属污染治理工程，逐步恢复沿河农田土地耕作功能。推进湿地公园、森林公园等建设，实施新一轮造林绿化、退耕还林等生态林业工程，全市森林覆盖率保持在65%以上。

放大生态优势。坚持走“生态产业化、产业生态化”的发展道路，构建绿色产业体系。实行严格的环境准入制，坚决不上高污染项目。通过特色园区建设、龙头企业带动、产业链条延伸等方式，发展生态农业、生态工业、生态服务业。改造提升传统产业，逐步将绿色技术、绿色工艺渗透到传统产业的各个环节；加快发展绿色新兴产业，培育新的绿色增长点。发展循环经济，推广资源节约替代、循环利用、零排放等技术。发挥公共机构“节能领跑、绿色发展”的示范引领作用。鼓励金融机构创新产品，加大对绿色产业的资金支持力度。

严格生态监管。坚持源头严防、过程严管、损害严惩、责任追究。把生态指标纳入社会发展综合评价体系，严格落实生态环境督办问责和离任审计制度，实行环境保护一票否决。强化环境综合治理，完善网格化监管，依法严厉打击环境违法行为。坚持“使用资源付费和谁污染谁治理、谁破坏生态谁付费”的原则，完善对重点生态功能区的生态补偿机制。

四、坚持改革开放，加快实现发展动力转换

未来五年，我们将坚持改革开放不动摇，大力实施创新驱动、开放带动，不断增强发展的活力。

以深化改革强动力。（1）继续简政放权，深化“放管服”改革，加快“三单一网”建设。（2）推进政府机构和事业单位制度改革，进一步理顺部门职责关系。（3）落实全面规范、公开透明的预算制度，实施跨年度预算平衡机制和中期财政规划管理。（4）理顺市以下收入划分，健全市、县两级事权与支出责任相适应的财政体制。（5）推进综合治税，加快实现税收治理体系和治理能力现代化。（6）深化城市管理体制改革，实现责、权、利相统一。（7）探索小型水利工程管理体制改革，创新管养机制、水资源产权制度和水权交易制度。（8）推进水、电力、交通、电信及自然资源等领域价格改革。（9）培育金融市场，激活民间融资，积极引进股份制银行、保险、证券、期货等金融机构，构建完善的金融市场体系。（10）进一步壮大国有投融资平台，不断放大资产效益，优化投资结构、提高融资能力，加强资金监管、做好风险防范。（11）深化国资国企改革，支持非公企业参与国企改革重组，推进国企产权多元化。（12）实施“五证合一、一照一码”，落实“先照后证”改革，推进电子营业执照和企业登记全程电子化。（13）整合统一规范的公共资源交易平台，实现交易全程电子化、公共资源“场外无交易”。（14）深化警务机制改革，实现向现代警务模式转型。

以创新创业增活力。依托国家创新型试点城市建设，构建特色区域创新体系，大力实施“八大科技创新”和“54321”工程，加快科技成果转化，加强知识产权创造与保护。完善“双创”扶持政策，引导风险投资、创业投资等资本投向“双创”领域，推动众创、众包、众扶、众筹等新业态发展。依托“互联网+”等新技术新模式，积极构建低成本、便利化、全要素、开放式的众创平台。鼓励各类孵化器和创业基地加快与互联网融合创新，推动创客空间、创新工场等新型众创空间发展。加速集聚“双创”领军人才，抓好拔尖人才、“百千万人才”等工作，完善统分结合、梯次衔接、政策配套的人才培养体系。

以对外开放扩大影响力。（1）树立开放大视野。放眼全省、全国乃至全世界，从世界经济联系中谋篇布局，在全球范围内配置资源，在更大范围、更宽领域、更高水平上推进对外开放。（2）构筑开放大平台。全面拓展与全球创意网络城市合作，提升“世界手工艺与民间艺术之都”影响力。全面融入“一带一路”和“长江经济带”，积极推动闽浙赣皖、赣东北等多边合作，推动人才、技术、资金、项目等各领域全方位开放。不断提升瓷博会“国际化、专业化、市场化、信息化、品牌化”水平，打造陶瓷文化的展示舞台、经贸合作的重要平台和对话世界的崭新名片。（3）全力实施大招商。把招商引资作为稳增长、扩投资的关键渠道来抓。充分发挥各级领导的带头示范作用，使招商工作氛围更浓、机制更全、推进更顺；积极参加并主办各类重大招商推介活动，推动招商引资向招商选资、精准招商转变；运用资本运作、股权投资等理念，发挥政府引导资金在招商引资中的引领放大作用；把握国内外产业转移趋势，重点围绕“3+1”产业，对接国内外知名企业，不断建链、强链、补链，促进产业壮大、链条齐全。吸引在外优秀人才返乡创业，鼓励已落户企业增资扩股。

五、坚持改善民生，切实增进百姓福祉

未来五年，我们将坚持以人为本，着力改善民生，促进和谐发展，不断增强发展的魅力。

扎实推进民生工程。深入实施精准扶贫，加大对贫困地区政策、项目、资金、人才支持力度，确保按期完成扶贫攻坚目标。完善就业创业培训服务体系，提高全社会就业水平。坚持居民收入增长与经济增长同步、劳动报酬提高与劳动生产率提高同步，多渠道增加城乡居民收入。以扩大保障覆盖面、提高保障标准为重点，稳步提升社会保障水平。加强社会救助制度与其他社会保障制度、专项救助与低保救助统筹衔接，构建综合救助工作格局。继续完善住房保障体系，力争享受住房保障政策家庭覆盖率达到23%以上。优先发展公共交通，完善公共交通网络。

协调发展社会事业。优化城乡教育布局，扩充优质教育资源，推动教育全面协调发展，基本实现教育现代化。加强“健康瓷都”建设，全面推进城市公立医院综合改革，建立健全分级诊疗制度，优化医疗卫生资源布局，促进医疗资源向基层、农村流动。完善重大疾病防控机制，科学实施重点疾病防治。稳妥实施全面“两孩”政策，综合治理出生人口性别比偏高问题。完善公共文化服务体系，创作一批具有鲜明地方特色和原创力的文化精品。大力发展群众体育和竞技体育，承办好第十五届省运会。大力发展老年医疗等健康服务业，促进“医养结合”，提高专业化养老服务水平。深入推进公民道德建设，弘扬诚信文化和法治精神，大力培育和践行社会主义核心价值观，不断提升市民文明素质。

全力维护社会稳定。推进依法治市，深化“平安瓷都”建设。严厉打击暴恐、黄赌毒、盗抢骗等违法犯罪活动，提升公众安全感。加强“数据强警”工程建设，依托大数据实现社会动态管控和精准防范。改进信访和调解工作，完善依法维权和矛盾化解体系。严格落实安全生产责任制，建立隐患排查治理动态监管体系，坚决预防和遏制重特大事故发生。加强食品药品和农产品质量安全监管，努力让人民群众吃得安全放心。加强防灾减灾体系建设，提升地震监测水平。完善城市应急管理体系，提高应急处置能力。做好征兵和双拥共建工作，加强国防和后备力量建设，深入贯彻军民融合发展战略，将国防动员工作融入全市经济社会发展体系。加强民族团结，切实维护民族宗教和谐稳定。

六、坚持实干为本，持之以恒抓好政府自身建设

空谈误国，实干兴邦。未来五年，我们将努力建设民生政府、责任政府、高效政府、法治政府、清廉政府，奋力开创政府工作新局面。

倡导创新思维。鼓励各级政府和广大干部解放思想、改革创新，抢抓机遇、奋发有为，勇于打破惯性思维，善于用合法合规又突破常规的办法破解难题。健全激励机制和容错纠错机制，支持创新、鼓励探索、宽容失败。

弘扬实干作风。从老百姓反映最强烈的问题抓起、最不满意的地方改起、最盼望的事情做起，及时回应老百姓的关切，不空谈、不观望、不扯皮，脚踏实地，担当实干，把既定的各项工作抓实、抓细、抓到位、抓出实效。

强化责任意识。对政府重点工作实行项目化管理，做到牵头领导、推进主体、配合单位、时序进度“四到位”，将挂在墙上、写在纸上的责任真正落到实处，做到“工作项目化、项目目标化、目标责任化、责任考评化”。

提升执行能力。“事不避难、义不逃责”。对于看准的事情、定下的决策，立说立行，马上就办，以最简的程序、最快的速度抓好落实，确保不误事、不掉链。

筑牢廉政底线。坚持依法行政、从严治政，严格按照法定程序、法定权限行使职权，将政府各项工作纳入法治轨道，做到权力事项清、权力流程清、权力责任清，有权必有责、用权受监督。从严落实党风廉政建设责任制，严肃查处以权谋私、失职渎职案件和腐败问题，切实做到干部清正、政府清廉。我们真诚地希望得到全市人民的监督。

各位代表，景德镇的发展已经迈上了新的长征路。大潮涌动，催人奋进；百舸竞发，不进则退。创新思路才能拓宽出路，干在实处才能走在前列！让我们高举中国特色社会主义伟大旗帜，紧密团结在以习近平同志为核心的党中央周围，在省委、省政府和市委的坚强领导下，带着对历史的敬畏之感，怀着对人民的赤子之情，初心不改，砥砺前行，努力开创景德镇各项事业新局面，为率先全面建成小康社会，把景德镇打造成一座与世界对话的城市而努力奋斗！
